# Host-Specific Circulation and Molecular Diversity of *Babesia*, *Hepatozoon* and *Bartonella* Species Among Wild Carnivores in Northwestern Italy

**DOI:** 10.3390/ani16192981

**Published:** 2026-09-22

**Authors:** Emily Sel, Barbara Moroni, Serena Robetto, Aitor Garcia-Vozmediano, Livia De Paolis, Emanuele Carella, Giulia Libertini, Ranieri Verin, Riccardo Orusa, Simona Zoppi, Elisabetta Razzuoli, Valeria Listorti, Lisa Guardone

**Affiliations:** 1 Department of Veterinary Science, University of Pisa, Viale delle Piagge 2, 56124 Pisa, Italy; emily.sel@phd.unipi.it (E.S.); giulia.libertini@vet.unipi.it (G.L.); ranieri.verin@unipi.it (R.V.); 2 Istituto Zooprofilattico Sperimentale del Piemonte, Liguria e Valle d’Aosta, Via Bologna 148, 10154 Torino, Italy; barbara.moroni@izsplv.it (B.M.); serena.robetto@izsplv.it (S.R.); aitor.garciavozmediano@izsplv.it (A.G.-V.); livia.depaolis@izsplv.it (L.D.P.); emanuele.carella@izsplv.it (E.C.); riccardo.orusa@izsplv.it (R.O.); simona.zoppi@izsplv.it (S.Z.); elisabetta.razzuoli@izsplv.it (E.R.); 3 Centro di Referenza Nazionale per le Malattie Degli Animali Selvatici (CeRMAS), Località Amerique 7G, 11020 Quart (AO), Italy

**Keywords:** vector-borne pathogens, tick-borne parasites, canids, mustelids, flea-borne pathogens

## Abstract

Vector-borne diseases (VBDs) represent a concern for animal and public health, and wildlife surveillance is valuable for their monitoring. The occurrence, molecular diversity and host distribution of tick-borne protozoa (TBP: *Babesia* spp., *Theileria* spp. and *Hepatozoon* spp.) and *Bartonella* spp. were investigated in wild carnivores from three regions of northwestern Italy. Spleen samples from 690 wild carnivores were screened for TBP and/or *Bartonella* spp. by PCR followed by sequencing. Among the 503 carnivores screened for TBP, 197 animals (39.2%) tested positive for at least one targeted protozoan species, including 87 positives for *Babesia* spp. (17.3%) and 120 for *Hepatozoon* spp. (23.9%). *Bartonella* spp. DNA was additionally detected in only one of the 363 animals tested (0.3%). Sequencing identified *Babesia* sp. badger types A and C in badgers, *B. vulpes* in foxes and *B. canis* in wolves, *Hepatozoon canis* in wolves and foxes, *H. martis* in stone martens and *Hepatozoon* sp. in stone and pine martens. Phylogenetic analyses supported the host-associated clustering of the detected lineages, confirming the molecular identification. These findings indicate the frequent occurrence of different TBP in wild carnivores from northwestern Italy, with marked host associated patterns, contributing to our understanding of VBD epidemiology with a One Health approach.

## Simple Summary

Wild carnivores provide valuable opportunities to monitor the circulation of vector-borne pathogens that may affect animal and human health. In this study, spleen samples from 690 wild carnivores (canids and mustelids) collected in northwestern Italy were screened for tick-borne protozoa (*Babesia*, *Theileria* and *Hepatozoon* spp.) and/or *Bartonella* spp. Tick-borne protozoa were frequently detected, whereas *Bartonella* spp. DNA was identified only in a single European badger. Molecular characterization revealed marked host-specific circulation patterns, with distinct *Babesia* and *Hepatozoon* species associated with different carnivore hosts. These findings provide an updated overview of the circulation of vector-borne pathogens in wild carnivores from northwestern Italy and highlight the value of wildlife surveillance for understanding pathogen ecology and supporting One Health disease monitoring.

## 1. Introduction

Vector-borne diseases (VBDs) are caused by a wide range of viruses, bacteria and parasites of increasing concern for both animal and public health [1]. In recent decades, the epidemiology of VBDs has been profoundly influenced by climate change, habitat fragmentation, landscape modification and the growing interface among wildlife, domestic animals and humans. These factors are known to contribute to changes in vector distribution, abundance and seasonal dynamics, favoring the spread of VBDs into previously non-endemic areas and increasing the risk of pathogen transmission to animals and humans [1,2,3,4,5].

Among VBDs, a relevant role is played by tick-borne diseases (TBDs) and tick-borne protozoa (TBP). *Babesia* spp., *Theileria* spp. and *Hepatozoon* spp. are apicomplexan TBP mainly transmitted by ticks that can infect a wide variety of domestic and wild vertebrates [6]. While *Theileria* species mainly infect ungulates, *Babesia* spp. infect a wide range of avian and mammalian hosts, such as domestic and wild carnivores, and some species also affect humans [7]. The life cycles of *Babesia* spp., *Theileria* spp. and *Hepatozoon* spp. involve asexual replication in vertebrate hosts and sexual development within ticks [8]. *Babesia* spp. and *Theileria* spp. are transmitted during tick feeding and infect red blood cells and/or leukocytes, whereas *Hepatozoon* spp. transmission generally occurs following ingestion of infected ticks and infect leukocytes and muscle fibers [9,10]. Several tick species have been implicated in the transmission of these protozoa in Europe, including *Ixodes ricinus*, *Rhipicephalus sanguineus* and *Hyalomma marginatum* for *Babesia* species [11], *R. turanicus*, *Dermacentor marginatus* and *H. lusitanicum* for *Theileria* spp. [12] and *R. sanguineus* for *Hepatozoon* spp. [10].

*Bartonella* spp. are Gram-negative, facultative intracellular bacteria that are highly adapted to mammalian hosts, capable of establishing long-lasting, often asymptomatic infections through effective immune evasion [13]. Their transmission primarily relies on blood-feeding arthropods, particularly fleas, which act as vectors, facilitating bacterial transmission both within and between host species. Several *Bartonella* species are zoonotic pathogens of veterinary and public health concern [13].

Wild carnivores are increasingly recognized as important hosts in the ecology of these pathogens. In Europe, *Babesia* spp. have been documented in several free-ranging carnivore species, including *B. canis* and *B. capreoli* in wolves (*Canis lupus*), *B. canis* and *B. vulpes* in red foxes (*Vulpes vulpes*), and badger-associated *Babesia* lineages in European badgers (*Meles meles*) and stone martens (*Martes foina*) [14,15,16]. Similarly, *H. canis* is widely distributed among wild canids, whereas other *Hepatozoon* species, such as *H. martis*, appear to be more closely associated with mustelids [17]. In contrast, reports of *Theileria* spp. in carnivores remain sporadic and are generally regarded as spill-over events with limited epidemiological significance [18]. In addition to TBPs, high prevalences of zoonotic *Bartonella* spp. and other arthropod-borne pathogens have recently been reported in wild carnivores from southern Italy, further supporting their potential role in the maintenance and circulation of VBPs at the wildlife–domestic animal interface [13].

Wild carnivores may also influence the epidemiology of TBP at the wildlife–domestic animal interface, with particular relevance for domestic dogs, which are susceptible to several *Babesia* and *Hepatozoon* species [19,20,21]. In dogs, these infections may range from asymptomatic carriage [20,22] to severe and potentially fatal disease if not promptly diagnosed and appropriately treated [23,24,25,26].

Over recent decades, several wild carnivore populations, particularly foxes and, more recently, wolves, have expanded across Europe, increasingly colonizing peri-urban and anthropized environments. Their growing abundance, ecological adaptability and potential interaction with domestic animals may favor the circulation and spread of TBP across different ecological settings. Consequently, wild carnivores represent valuable sentinels for monitoring the circulation of agents of VBDs, which is essential for improving risk assessment for both domestic animals and public health [4,27].

Despite this, information on the occurrence and molecular diversity of apicomplexan TBP and *Bartonella* spp. in wild carnivores from northwestern Italy remains limited, and their potential role in pathogen transmission at the wildlife–domestic animal interface is still poorly understood. This geographical area supports abundant and diverse populations of wild carnivores, including red foxes, Italian wolves, and several mustelid species. Therefore, the present study aimed to investigate the occurrence, molecular diversity and host distribution of *Babesia* spp., *Theileria* spp., *Hepatozoon* spp. and *Bartonella* spp. in wild carnivores from northwestern Italy.

## 2. Materials and Methods

### 2.1. Sampling

Wild carnivores found dead between 2022 and 2026 in Piemonte, Liguria and Valle d’Aosta and submitted to the Istituto Zooprofilattico Sperimentale del Piemonte, Liguria e Valle d’Aosta (IZSPLV) through the regional passive surveillance system were considered for inclusion in the study. The target species comprised two canids, the wolf (*Canis lupus*) and red fox (*Vulpes vulpes*), and four mustelids, the European badger (*Meles meles*), stone marten (*Martes foina*), pine marten (*Martes martes*) and Eurasian otter (*Lutra lutra*). Carcasses were recovered by veterinary health services or other competent local authorities (e.g., regional wildlife surveillance units) following notification, and transported to the competent IZSPLV territorial section for post-mortem examination. Animals were eligible for inclusion when they belonged to the target wild carnivore species (canids and mustelids), originated from one of the three study regions, were collected within the study period, and had spleen tissue available and suitable for molecular analysis. No animals were killed specifically for the purposes of this study.

Carnivores underwent post-mortem examination and sampling according to routine diagnostic procedures, after storage at +4 °C (for maximum 24 h) or at −20 °C (up to one month of storage). Spleen samples were collected from eligible animals, and information on host species, sex, estimated age and geographical origin was recorded whenever available. Age was estimated based on dental characteristics and body size evaluation [28,29]. Spleen samples analyzed for TBP were subsequently processed and molecularly tested at the Laboratory of Parasitology, Department of Veterinary Sciences, University of Pisa, while molecular analyses for *Bartonella* spp. were performed at the Turin branch of the IZSPLV. All samples were stored at −20 °C until molecular processing and analysis. In addition, from a small subset of foxes from Liguria (five fresh carcasses, collected <24 h after death and not frozen), approximately 2 mL of blood was collected aseptically from the cardiac chambers using a sterile 5 mL syringe for cytological evaluation (see Section 2.4).

### 2.2. DNA Extraction and Molecular Analysis

Animals were screened for TBP (*Babesia*/*Theileria* spp. and *Hepatozoon* spp.), *Bartonella* spp., or both, according to the molecular investigations performed. Consequently, the study comprised partially overlapping subsets of animals analyzed for TBP (*n* = 503), *Bartonella* spp. (*n* = 363), or both (*n* = 176). Therefore, pathogen-specific denominators were used throughout the study for prevalence estimation.

For protozoa, DNA extraction was performed from approximately 15 mg of spleen tissue using the DNeasy Blood & Tissue Kit (QIAGEN, Hilden, Germany) according to the manufacturer’s instructions. For *Bartonella* spp., approximately 30 mg of tissue was homogenized in tubes containing zirconium oxide beads, after the addition of 300 µL of phosphate-buffered saline (PBS). DNA extraction was then performed using the Promega RSC Blood DNA kit (Promega, Madison, WI, USA) and with the Maxwell RSC 48 automated extraction system (Promega, Madison, WI, USA), according to the manufacturer’s instructions. Extracted DNA samples were stored at −20 °C until molecular analysis. Samples were screened for *Babesia*/*Theileria* spp. and *Hepatozoon* spp. by conventional end-point PCR targeting the 18S rRNA gene using previously published protocols, while *Bartonella* spp. DNA was tested by real-time PCR. Details of primers, target genes, PCR amplification conditions, and expected amplicon sizes are reported in Table 1.

Sterile molecular-grade water was included as a negative control in each PCR assay, whereas DNA previously confirmed positive samples of *Babesia*/*Theileria* species, *Hepatozoon* spp. and *Bartonella* spp. served as positive controls. Amplifications for TBPs were performed using Wonder Taq DNA Polymerase (Euroclone, Milan, Italy) and a T100^TM^ Thermal Cycler (Bio-Rad, Hercules, CA, USA). PCR products were visualized using a UV transilluminator after electrophoresis migration on 2% agarose gel stained with GelRed^®^ Nucleic Acid Gel Stain (Biotium, Fremont, CA, USA) in 1× standard tris-acetate-EDTA (TAE) buffer. SharpMass™ 100 Plus Ladder (Euroclone, Milan, Italy) was used as a molecular weight marker. Amplicons of the expected fragment size were considered positive. Representative positive amplicons were purified and subjected to Sanger sequencing by Eurofins Genomics (Ebersberg, Germany). Consensus sequences were compared with reference sequences available in GenBank using the BLAST algorithm (program version: BLASTN 2.17.0+) (http://www.ncbi.nlm.nih.gov/BLAST accessed on 6 July 2026). PCR for *Bartonella* spp. was carried out using the TaqMan Probe 2× qPCR Super Mix UDG V5 kit (Fisher Molecular Biology, Trevose, PA, USA); amplification and fluorescence detection were performed using a CFX96 Touch™ Real-Time PCR Detection System (Bio-Rad, Hercules, CA, USA).

### 2.3. Phylogenetic Analysis

Successfully obtained sequences were aligned before GenBank deposition and phylogenetic analysis. Sequences showing 100% nucleotide identity were grouped, and one or more sequences per group were retained to preserve representation of host species and geographical origin, whereas sequences showing distinct nucleotide patterns were retained. This approach reduced redundancy while maintaining the molecular, host and geographical diversity of the detected genotypes. Overall, 39 sequences were deposited in GenBank (accession numbers PZ805153-PZ805159, PZ898538-PZ898567, PZ957578-PZ957579). The same sequences were used for the phylogenetic analysis, together with reference sequences selected from GenBank on the basis of BLAST similarity and with sequences previously reported from different host species and geographical areas.

Separate phylogenetic trees were reconstructed for *Babesia* spp. and *Hepatozoon* spp. based on partial 18S rRNA gene sequences using the maximum likelihood (ML) method implemented in MEGA12 [33]. Evolutionary distances were estimated using the Tamura-Nei nucleotide substitution model [34]. Node support was assessed by bootstrap analysis with 1000 replicates, and only bootstrap values ≥ 70% are shown. *Plasmodium falciparum* (GenBank accession JQ627152) and *Babesia equi* (GenBank accession LC008132) were used as outgroups for *Babesia* and *Hepatozoon* phylogenies, respectively.

### 2.4. Cytological Analysis

Immediately after collection, intracardiac blood samples were used to prepare six thin blood smears per animal on clean glass slides. As mentioned (Section 2.1), cytology was only performed on a subset of five fresh fox carcasses. The air-dried smears were stained using a May–Grünwald–Giemsa Quick Stain kit (MGG Quick Stain, code 04-090805; Bio-Optica Milano S.p.A., Milan, Italy), according to the manufacturer’s instructions, and examined using a Leica DM1000 LED light microscope (Leica Microsystems, Wetzlar, Germany). For each smear, ten representative, non-overlapping fields within the monolayer area were examined using 20× and 40× objectives; the field area at 40× was 0.237 mm^2^. Thus, 60 fields per animal were evaluated at each objective magnification by a board-certified pathologist (RV). The cytological assessment was qualitative and included erythrocyte morphology, the relative representation and morphology of leukocyte populations, and the presence and morphology of hemoparasites. No formal erythrocyte count or quantitative estimation of parasitemia was performed.

### 2.5. Statistical Analysis

Statistical analyses were performed using R software version 4.5.3 (R Foundation for Statistical Computing, Vienna, Austria). Descriptive statistics were used to summarize host species, sex, age class and geographical origin. Apparent prevalence was calculated as the proportion of PCR-positive animals among those tested for each target pathogen, and exact binomial 95% confidence intervals (95% CIs) were estimated using the Clopper–Pearson method.

Owing to the limited number of individuals available for some mustelid species and the resulting sparse outcome distributions, multivariable inferential analyses, limited to *Babesia* spp. and *Hepatozoon* spp., were restricted to canids (wolves and foxes), whereas results for mustelids were summarized descriptively. In canids, unadjusted associations between pathogen positivity and host species, region, sex and age were initially explored using Pearson’s chi-square test or Fisher’s exact test, as appropriate for sparse contingency tables. These analyses were considered exploratory and were not used as a statistical screening criterion for inclusion in the multivariable models.

Separate multivariable logistic regression models were fitted for *Babesia* spp. and *Hepatozoon* spp. Host species, region of origin, sex and age category were selected *a priori* as explanatory variables on the basis of their biological and epidemiological relevance and were included simultaneously in both models. Because the original age classification contained sparse categories, age was recategorized as “young” (including cubs, juveniles and subadults) or “adult”. Sparse outcome distributions, including zero-positive cells in some host-region strata, could result in unstable conventional maximum likelihood estimates; therefore, Firth’s penalized likelihood logistic regression was applied to obtain bias-reduced parameter estimates, while considering complete cases. Animals with missing information for one or more covariates included in the models were excluded, and no imputation of missing data was performed. Results were expressed as adjusted odds ratios (OR_adj_) with corresponding 95% confidence intervals. For all analyses, statistical significance was set at *p*-value < 0.05.

## 3. Results

### 3.1. Prevalence of Target Pathogens

A total of 690 wild carnivores were collected during the study period, comprising 349 foxes, 175 wolves, 132 badgers, 28 stone martens, five pine martens and one otter (Table 2). Most animals originated from Piemonte (*n* = 459), followed by Valle d’Aosta (*n* = 151) and Liguria (*n* = 80), encompassing 344 municipalities across northwestern Italy (Figure 1). Species composition differed among regions, with wolves mainly represented in Piemonte and Liguria, whereas foxes accounted for most carnivores collected in Valle d’Aosta.

Among the 503 carnivores screened for TBPs, 197 (39.2%; 95% CI: 34.9–43.6) tested positive for at least one of the targeted protozoa. Overall, 87 animals were positive for *Babesia* spp. and 120 for *Hepatozoon* spp., including 10 foxes coinfected with both pathogens. In contrast, *Bartonella* spp. DNA was detected in only one of the 363 animals tested (0.3%; 95% CI: 0.01–1.5), corresponding to a single European badger.

Distinct host-associated circulation patterns emerged for the two protozoa (Table 3). *Babesia* spp. DNA was mainly detected in badgers and foxes, with prevalences of 37.4% (95% CI: 28.2–47.3) and 18.8% (95% CI: 13.8–24.6%), respectively, whereas wolves were only occasionally positive (3.9%; 95% CI: 1.5–8.4). Conversely, *Hepatozoon* spp. predominated in wolves (46.1%; 95% CI: 37.9–54.3), followed by foxes (21.1%; 95% CI: 15.9–27.1), while positivity among mustelids was sporadic and limited to three stone martens and one pine marten. No badger tested positive for *Hepatozoon* spp., whereas all stone martens, pine martens and the otter were negative for *Babesia* spp. No *Theileria* spp. DNA was detected in the spleen samples examined.

These host-associated differences were also supported by multivariable analysis (Table 4). Foxes showed markedly higher odds of *Babesia* positivity than wolves (OR_adj_ = 6.16; 95% CI: 2.55–17.70), whereas they exhibited lower odds of *Hepatozoon* positivity (OR_adj_ = 0.41; 95% CI: 0.24–0.70). Compared with animals from Piemonte, those from Valle d’Aosta showed lower odds of positivity for both *Babesia* spp. (OR_adj_ = 0.08; 95% CI = 0.01–0.31) and *Hepatozoon* spp. (OR_adj_ = 0.31; 95% CI = 0.11–0.75), whereas no differences were detected between Liguria and Piemonte. Detailed results according to host species and region are reported in Table S1. Sex was not associated with either *Babesia* spp. or *Hepatozoon* spp. positivity. Age was also not associated with *Babesia* spp. positivity, whereas adults seemed to show lower adjusted odds of *Hepatozoon* positivity than young animals, although the confidence interval included the null value (OR_adj_ = 0.61; 95% CI = 0.37–1.01).

### 3.2. Sequencing Results

Among the positive samples, 44 *Babesia* spp. amplicons (50.6% of positive samples) and 58 *Hepatozoon* spp. amplicons (48.3%) were successfully sequenced. Sequencing was performed on 19 samples from badgers, 21 from foxes and four from wolves for *Babesia* spp., and on 35 samples from wolves, 19 from foxes, three from stone martens and one from pine martens for *Hepatozoon* spp.

Sequencing analysis supported marked host-associated circulation patterns. Phylogenetic reconstruction clearly separated canid-associated and badger-associated *Babesia* lineages (Figure 2), supporting the host-associated distribution observed in the prevalence analysis. Among badgers, two *Babesia* lineages were identified. In particular, 13 sequences showed 99.67–100% similarity with *Babesia* sp. badger type C previously identified in China (GenBank: MG799847) and in badgers from the Czech Republic (GenBank: OQ816786; [35]), whereas six sequences showed 99.74–100% similarity with *Babesia* sp. *badger* type A described in China (GenBank: MG799845), the United Kingdom (GenBank: MT052179; [36]) and Spain (GenBank: KT223484).

All sequenced fox-derived *Babesia* isolates clustered within the *B. vulpes* clade and showed complete identity with sequences previously described in wild carnivores from Spain (GenBank: KT223483; [7]), as well as with *B. vulpes* isolates reported from Russia (GenBank: MT509981), southern Italy (GenBank: PX387919; [37]), and the Czech Republic (GenBank: OQ816785; [35]). The sequences were also identical to the *Babesia* sp. “Spanish dog” genotype (syn. *B. vulpes*) (GenBank: AY457974), originally described by Camacho et al. [38]. Conversely, all wolf-derived sequences clustered with *B. canis*, showing high similarity (99.57–100%) with isolates previously identified in Iberian wolves (*Canis lupus signatus*) from northwestern Spain (GenBank: PP574310; [39]), and with sequences previously identified in dogs (PQ108690), *Ixodes ricinus* ticks from Poland (GenBank: KR003829; [40]) and horses from northwestern Italy (GenBank: KX839230; [41]).

Sequencing of *Hepatozoon* spp. amplicons also revealed clear host-related patterns (Figure 3). All sequenced *Hepatozoon*-positive wolf samples showed 99.28–100% similarity with *H. canis* isolates previously reported in domestic and wild canids from several European countries (GenBank: PQ001799, OP012802, MH699889, AY461375, KF322141, KT215366, KM096412, MK757793, MK757784, MN103412, MG254576, MK757781, MK757770, OR282485). Similarly, the 19 sequenced fox samples showed complete identity with *H. canis* sequences available in GenBank (GenBank: OP012783, PX467462, MG254591, KX879141, MT107096, MH699889, KF322144, MK757784). By contrast, *Hepatozoon*-positive mustelids formed a separate cluster comprising *H. martis* and another mustelid-associated *Hepatozoon* lineage. Two stone marten sequences showed 100% identity with *H. martis* (GenBank: OM256566), while a third matched *Hepatozoon* sp. (GenBank: OM256569), both previously reported in martens from Hungary [42]. The sequence obtained from the single positive pine marten (GenBank: PZ898565) was considerably shorter than the expected PCR product (152 bp compared with approximately 660 bp) and showed 93.14% nucleotide identity with *H. martis* (GenBank: OR144421). Although it showed phylogenetic affinity with *H. martis*, its placement was weakly supported (bootstrap value 29%). Given the limited sequence length, relatively low nucleotide identity and weak phylogenetic support, confident species-level assignment was not possible. Sequencing additionally confirmed co-infections with *B. vulpes* and *H. canis* in all ten fox samples positive for both PCR assays.

The single *Bartonella* spp. positive sample was further investigated by sequencing. However, after three independent attempts, sequencing of the PCR amplicon was unsuccessful, preventing further molecular characterization.

### 3.3. Cytology

Cytological abnormalities were identified in one of the five foxes examined. The affected animal was a *Babesia vulpes*-PCR-positive red fox. The blood smear showed a subjectively reduced erythrocyte population, associated with moderate-to-marked regenerative morphological changes characterized by prominent polychromasia and moderate anisocytosis. Numerous erythrocytes were hypochromic, and occasional spherocytes were observed. Numerous erythrocytes contained one or more small (1–2 µm), intracellular signet ring-shaped piroplasms with a single eccentrically positioned, densely basophilic nucleus (Figure 4). Rare nucleated erythroid precursors were also identified. Leukocytes appeared moderately increased in relative representation within the examined fields and consisted predominantly of mature, segmented neutrophils, accompanied by moderate numbers of lymphocytes and lower numbers of band neutrophils and monocytes. These findings represent semiquantitative cytomorphological observations rather than quantitative hematological measurements. No relevant cytological abnormalities were identified in blood smears from the remaining four foxes, which were PCR-negative.

## 4. Discussion

The present study provides updated molecular evidence on the occurrence and diversity of *Babesia* spp., *Hepatozoon* spp. and *Bartonella* spp. in wild carnivores across a large area of northwestern Italy, where available information has been previously fragmented or limited. The findings revealed marked host-associated patterns, with badgers and foxes accounting for most *Babesia* spp. detections, wolves showing the highest prevalence of *Hepatozoon* spp., and *Bartonella* spp. being detected in only one badger.

The highest prevalence of *Babesia* spp. was observed in badgers (37.38%), followed by foxes (18.81%) and wolves (3.95%). Three badger-associated *Babesia* genotypes—types A, B and C—have been reported in the literature. In the present study, type C was the most frequently detected, followed by type A, whereas type B was not found. The distribution differs partially from that reported in a recent study involving nine continental European countries (Austria, Bosnia and Herzegovina, Croatia, France, Germany, Hungary, Italy, Romania, and Serbia), where type A was the most prevalent genotype, followed by type C and type B [16]. High prevalences of *Babesia* sp. badger type A have also been reported in Spain [15], the United Kingdom [36,43] and, more recently, in Hungary [44], and this genotype has previously been identified in both northern and southern Italy [2,14]. By contrast, records of *Babesia* sp. badger type C are largely restricted to southern Italy and eastern European countries [16]. Although type B was not identified in our study, it has been reported in Spain [7], Scotland [43] and in northern Italy [2]. Badger-associated *Babesia* lineages have also been reported in wolves, wildcats, and domestic dogs in Europe, suggesting that these genotypes may not be strictly restricted to badgers and warranting continued investigation of their host range and veterinary relevance [16].

The prevalence of *B. vulpes* in foxes varies considerably across Europe, ranging from approximately 26% to more than 60% [9,15,45,46,47,48]. Marked geographical variation has also been documented in Italy, with prevalences ranging from 0.98% to 10.34% in northern regions [2,11], 1.60% in central Italy [3] and 42.60% in southern Italy [14]. The prevalence observed in the present study (18.81%) therefore falls within the wide range previously reported at both national and European levels. Within the study area, positivity differed among regions, being highest in Liguria (33.33%), followed by Piemonte (23.97%) and Valle d’Aosta (1.75%). The markedly lower odds observed in Valle d’Aosta after accounting for host species, sex and age, suggest that the geographical pattern was not explained solely by differences in these host characteristics. Nevertheless, local ecological conditions, vector distribution, surveillance intensity and sampling structure may all have contributed to the observed differences.

The frequent detection of *B. vulpes* in foxes is consistent with previous evidence indicating that red foxes are important wildlife hosts for this parasite. This finding is also relevant for domestic animal health, as *B. vulpes* can infect dogs [20], and the first autochthonous clinical case in Italy was reported in 2025 [24]. Cytological examination was possible only for a very limited subset of animals (*n* = 5), because most carcasses had been frozen before necropsy and/or were already affected by post-mortem changes. In one *B. vulpes*-PCR-positive fox, intraerythrocytic piroplasms and erythrocyte morphological changes suggestive of a regenerative response were observed. Generally, molecular methods are considered to have higher analytical sensitivity, as previously reported from dead foxes [49]. Conversely, greater agreement between blood-smear examination and PCR may be expected during acute clinical infection, when parasitemia is higher, as recently observed in live foxes admitted to a wildlife rescue center [50]. Considering the small number of animals analyzed by cytology in our study, no comparison can be made between molecular and cytology diagnostic performance and no conclusions can be drawn on this aspect.

Another relevant *Babesia* species which we exclusively found in wolves was *B. canis.* The observed prevalence (3.95%) was comparable with values reported in wolves from northern Italy (0.9%), Spain (3.1%) [1], and Croatia (5.5%) [51], although substantially higher prevalences have been recorded in Poland (18.0%) [52] and Lithuania (30.0%) [10]. *Babesia canis* is the major cause of clinical canine babesiosis in Europe [53] and its detection in wolves is therefore of potential relevance when considering pathogen occurrence at the wildlife–domestic animal interface. Although infection appears to be less frequently associated with clinical disease in wolves than in domestic dogs, suggesting greater tolerance or adaptation in the wild host [51], clinical cases have been described, with signs including pyrexia, lethargy, anorexia, behavioral alterations, and hematocyturia [54]. The expanding distribution of wolves and their increasing use of peri-urban environments may enhance opportunities for sharing infected vectors with domestic dogs, although the actual epidemiological contribution of wolves to transmission cycles remains to be clarified.

No *Babesia* spp. were detected in stone or pine martens. Available information for these hosts remains limited. In stone martens, *B. vogeli* [55] and *Babesia* sp. badger type A [15] have previously been reported in Spain. To our knowledge, confirmed *Babesia* infection has not been reported in pine martens [15]. Given the small number of pine martens examined, however, the absence of detection should not be interpreted as evidence that this host is not susceptible.

A contrasting host-associated pattern emerged for *Hepatozoon* spp. *Hepatozoon canis* was detected in 46.05% of wolves and 21.10% of foxes, and wolves had significantly higher adjusted odds of positivity than foxes. Previous European surveys on wolves have reported prevalences ranging from 35.0% to 93.0% in Poland [56], Croatia [57], Serbia [58], Lithuania [10], Portugal and Spain [1]. In Italy, prevalences of 75.7% [2] and 92.0% [1] have been reported in the same broad geographical area. The prevalence observed here was therefore lower than previous Italian estimates but remained within the wider European range. For foxes, prevalences reported across Europe have generally been higher than that detected in the present study (21.10%), ranging approximately from 38% to 92% [9,45,46,47,48,57,59,60], although a markedly lower prevalence of 5.1% was previously reported in Piemonte [2]. These differences may reflect variation in host ecology, local vector abundance, infection pressure, sampling design or the characteristics of the populations examined. Unlike other TBP, *H. canis* is transmitted when a vertebrate host ingests ticks containing mature oocysts rather than through the tick bite itself [21]. In dogs, clinical severity is influenced by parasite burden, and high parasitemia may be associated with fever, lethargy, anemia, cachexia and, in severe cases, death [61]. The frequent detection of *H. canis* among the examined wolves and foxes indicates frequent infection in the sampled wild canids. However, implications for domestic dogs depend on the local distribution and infection prevalence of competent tick hosts, which were not included in the present study. The observed epidemiological patterns were therefore mainly associated with host species and geographical origin, whereas demographic differences were comparatively modest.

Among mustelids, *H. martis* and another not-yet-identified *Hepatozoon* sp. were detected in stone martens. *Hepatozoon martis* has previously been reported in stone martens from Spain [9], Croatia [62], Czech Republic [35], and Hungary [42], with prevalences ranging from 33% to 81%. In Italy, molecular and histopathological evidence of this species was reported only recently in five out of 15 tested animals examined by qPCR assay [63]. An unidentified *Hepatozoon* sp. has also been reported in stone martens, with a prevalence of 33.33% [42], and closely related lineages have been described in an American mink in the United States [64] and in a raccoon in Czech Republic [35]. *H. canis* has also been reported in stone martens [9], but it was not detected in this host in our study. The observed prevalences of *H. martis* (8.7%) and the unidentified *Hepatozoon* sp. (4.3%) were lower than those reported previously, potentially because of the limited sample size, geographical variation, or differences in vector exposure. Nevertheless, the molecular findings document the occurrence of multiple mustelid-associated *Hepatozoon* lineages in northwestern Italy.

The single positive pine marten carried a sequence related to *H. martis*, although its relatively low similarity and limited sequence quality precluded confident species assignment. *Hepatozoon* spp. have been reported in pine martens [65,66], including a prevalence of 100% for *H. martis* in a small study (*n* = 10 individuals) conducted in Bosnia and Herzegovina [62].

No badgers tested positive for *Hepatozoon* spp. Only a few studies have investigated these parasites in European badgers. Published prevalences vary widely, from 1.6% in Croatia [57] to 75% in Hungary [42] for *H. martis*, while *H. canis* was detected in approximately 7.8% of Croatian badgers [57]. The absence of positive animals in the present study may reflect genuinely low occurrence in the study area, differences in vector exposure or limitations associated with detecting rare infections. To date, information on *Hepatozoon* infection in badgers from Italy remains scarce.

No *Theileria* spp. DNA was detected in the spleen samples examined. This finding is consistent with a study involving 285 wolves from Spain, Italy and Portugal in which *Theileria* spp. were not detected [1] and with the general sporadic occurrence of *Theileria* spp. reported in wild carnivores [18]. Nevertheless, molecular detections have been reported elsewhere in Europe. Beck et al. [57] identified *Theileria capreoli* and *Theileria* sp. 3185/02 in 15 out of 107 wolves from Croatia, with sequences identical to those isolated in roe and red deer, while *Theileria* sp. 3182/05, a parasite frequently reported in wild cervids, was detected in a single fox in Croatia [67]. The PCR target and primers used in our study [30] were the same as those applied by Beck et al. [57], supporting their suitability for detecting *Theileria* DNA. However, the absence of molecular detection in the examined animals cannot be interpreted as evidence that *Theileria* infection is absent from the study area. Low pathogen occurrence, differences in host or vector exposure and matrix-dependent detectability may all have contributed to the negative findings. Further information on *Theileria* spp. occurrence in local wild ruminants and vectors would be useful for interpreting these results [11].

As already discussed, from a One Health perspective, the results highlight the occurrence of TBP of recognized veterinary importance in the studied wildlife, such as *B*. *canis*, *B*. *vulpes* and *H*. *canis*, which are associated with possible severe infections in dogs [68]. Additionally, although the clinical relevance of TBP in wildlife is poorly understood, *B. canis* was believed to have caused a fatal acute disease in a wolf in Hungary and in Croatia (reported in [3]) and was identified as responsible for a clinical case in a farmed wolf in Poland [54], so their impact in wildlife conservation should not be underestimated. Although the most relevant zoonotic species of *Babesia* in Europe (*B. divergens* and *B. microti*) were not detected in the present study, the zoonotic species *B. venatorum* and *B. microti* were found in foxes in Germany [69] and *B. canis* and *B. vulpes* were reported in two human cases in North-west Italy [70].

An additional finding of interest was the occurrence of *Babesia*–*Hepatozoon* co-infections in foxes. Interestingly, nine of the ten foxes showing co-infection with *B. vulpes* and *H. canis* originated from Piemonte. This apparent geographical clustering may reflect differences in sampling intensity, host ecology, vector exposure or local environmental conditions. However, given the limited number of co-infected animals and the uneven distribution of foxes among regions, this pattern should be interpreted cautiously. Co-infections with *B. vulpes* and *H. canis* have also been frequently reported in foxes across Europe [9,45,46]. This finding indicates that foxes may be simultaneously exposed to multiple vector-borne protozoa and may reflect overlapping transmission cycles or shared ecological and vector-related exposure. To our knowledge, no studies reported synergistic pathogenicity or competition between these two pathogens, but spleen and kidney enlargement were observed in co-infected individuals in Poland [46].

In this study, where possible, ticks were collected from the same animals. Collected data are still being elaborated, however preliminary analyses [71] showed strong association with *Ixodes ricinus*, the most widespread tick species in the wild in Europe, reinforcing the broad ecological distribution of this species across both ungulate and carnivore hosts. In contrast, *Ixodes hexagonus* was predominantly associated with mesocarnivores, particularly red foxes and badgers. While *I. ricinus* has been reported as a vector of several *Babesia* spp. [11], *I. hexagonus* is the proposed main vector of *B. vulpes*, and its high prevalence was related to the high prevalence of *B. vulpes* (formerly *T. annae*) in Spain [72].

Only one animal, a European badger from Piemonte, tested positive for *Bartonella* spp. Given this single detection, no inference and no conclusions can be drawn; therefore, the epidemiological significance of this finding remains uncertain. Nevertheless, the detection is noteworthy because, to our knowledge, previous Italian wildlife surveys have not reported *Bartonella* spp. DNA in wild badgers. Previous studies in northern Italy reported *Bartonella* spp. prevalence values above 7% in red foxes found dead in Piemonte and Valle d’Aosta [73], while higher values have been described in central and southern Italy among wolves, foxes, hedgehogs, and roe deer, reaching up to 83% in wolves, although this estimate was based on only six animals [13]. In the present study, none of the wolves examined tested positive. However, direct comparison among studies should be made cautiously because differences in host composition, geographical setting, flea communities, vector exposure, diagnostic protocols or sampling design may substantially influence detection frequencies. Several *Bartonella* species previously identified in Italian wildlife, including *B. schoenbuchensis*, have zoonotic relevance [13], emphasizing the importance of molecular characterization. In the present study, repeated sequencing attempts were unsuccessful, preventing species-level identification and, consequently, any assessment of the zoonotic potential of the detected organism.

The pathogenic and epidemiological roles of *Bartonella* spp. in wildlife remain poorly understood. Nevertheless, *Bartonella* spp. have been detected in several wild carnivores, indicating that these hosts may contribute to natural transmission cycles [55,62,74,75,76,77]. Fleas are considered important vectors of several *Bartonella* species and commonly infest wild carnivores, potentially facilitating transmission among susceptible sympatric hosts [78]. Data on *Bartonella* spp. circulation in Italian domestic animals are also limited, with one serological survey reporting 6% positivity for *B. henselae* in owned dogs from Tuscany [79]. In light of the single uncharacterized detection obtained in the present study, further investigations incorporating larger and appropriately designed host samples, ectoparasite screening, and molecular characterization are needed to determine whether *Bartonella* spp. are consistently maintained in wild carnivore populations and to clarify their potential relevance at the wildlife–domestic animal interface.

Several limitations should be considered when interpreting the findings of the present study. Firstly, the animals originated from passive surveillance of found-dead wildlife, thus not necessarily representing the wild population, and were unevenly distributed among host species and regions. Consequently, inclusion depended on carcass detection, recovery and submission to the surveillance system, rather than on probability-based sampling of living wild populations. This may have introduced selection bias, as animals in poorer health conditions or affected by conditions increasing mortality could be disproportionately represented. Such selection may lead to overestimation of infections associated with morbidity or mortality, whereas infections occurring predominantly in apparently healthy individuals or in animals less likely to be detected and recovered could be underestimated. Accordingly, the apparent prevalence estimates primarily described the examined carcass-based population and should not be directly extrapolated to the wider living wild carnivore population of northwestern Italy. Likewise, pathogen detection should not be interpreted as evidence that infection contributed to the animal’s death. A further limitation concerns the use of spleen tissue as the primary matrix for molecular screening, while blood smears were available only for a very limited subset of animals. Pathogen detectability may vary among biological matrices, as previously demonstrated for *Babesia* spp. and *Hepatozoon* spp. in wild canids [47,50,80]. Therefore, infections characterized by low parasite burden or limited presence in splenic tissue may have remained undetected, potentially leading to underestimation of pathogen occurrence. Similarly, reliance on a single tissue matrix may have limited the diversity of lineages detected. Moreover, the small number of individuals available for some mustelid species limited statistical comparisons, while partially overlapping subsets of animals were tested for TBP and *Bartonella* spp., requiring the use of pathogen-specific denominators. In addition, sequencing was performed on representative subsets of positive amplicons, and the only *Bartonella*-positive sample could not be molecularly characterized. Finally, due to practical constraints it was possible to perform cytology only on a small subset of animals (five fresh fox carcasses from Liguria) and not on all positive animals; therefore, no comparison can be made between molecular and cytological diagnostic performance. Despite these constraints, the large number of animals examined, the broad geographical coverage and the integration of prevalence estimates, multivariable analyses and phylogenetic characterization provide a valuable overview of the circulation and molecular diversity of the targeted VBPs in wild carnivores from the study area.

## 5. Conclusions

The present study provides updated epidemiological and molecular evidence on the occurrence of *Babesia* spp., *Hepatozoon* spp. and *Bartonella* spp. in wild carnivores from northwestern Italy. Although research findings are specific to northwestern Italy, broader European comparisons indicate that observed patterns are consistent with surrounding areas. Marked host-associated patterns were observed, with *B. vulpes* predominantly detected in foxes, *B. canis* in wolves, and *Babesia* sp. badger type A and type C in badgers, whereas *H. canis* was mainly identified in wild canids, and *H. martis* and other *Hepatozoon* sp. lineages were identified in mustelids. To our knowledge, the detection of *H. martis* represents the first molecular evidence of this parasite in northwestern Italy. By contrast, *Bartonella* spp. DNA was detected only in a single European badger and could not be further characterized, so no epidemiological conclusions can be drawn and future research is needed to investigate the significance of this finding.

Overall, these results provide a regional baseline for the occurrence and molecular diversity of vector-borne pathogens in wild carnivores and highlight the value of passive wildlife surveillance for documenting pathogen ecology at the wildlife–domestic animal interface. Further integrated studies including wildlife hosts, domestic animals and arthropod vectors will be required to clarify transmission dynamics and assess the epidemiological relevance of the detected pathogens within a One Health framework.

## Figures and Tables

**Figure 1:**
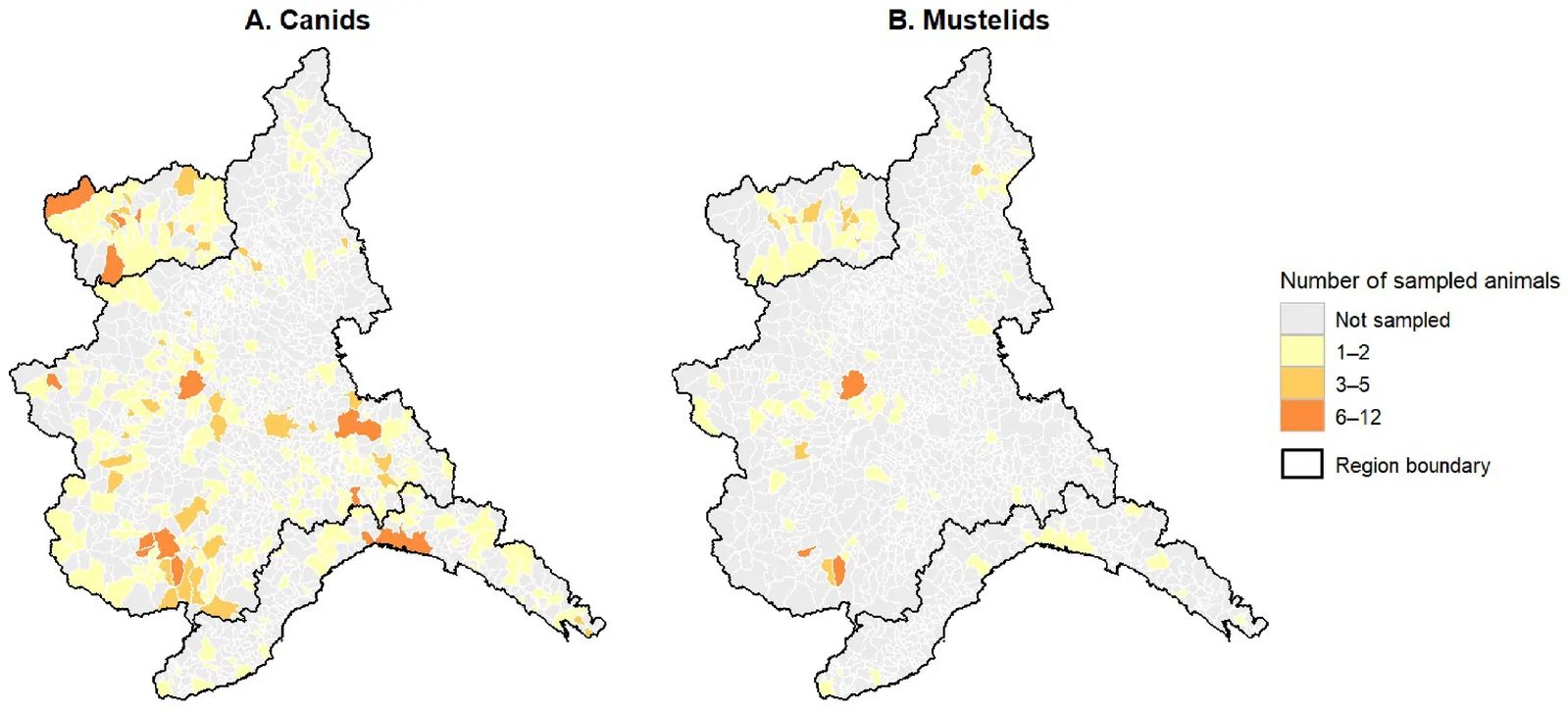
Geographic distribution of sampling locations for wild carnivores included in the study. Municipality-level distribution of sampled: (**A**) canids (*Canis lupus* and *Vulpes vulpes*); (**B**) mustelids (*Meles meles*, *Martes foina*, *Martes martes* and *Lutra lutra*) collected in northwestern Italy between 2022 and 2026. Municipality shading represents the number of sampled animals according to Jenks natural breaks classification.

**Figure 2:**
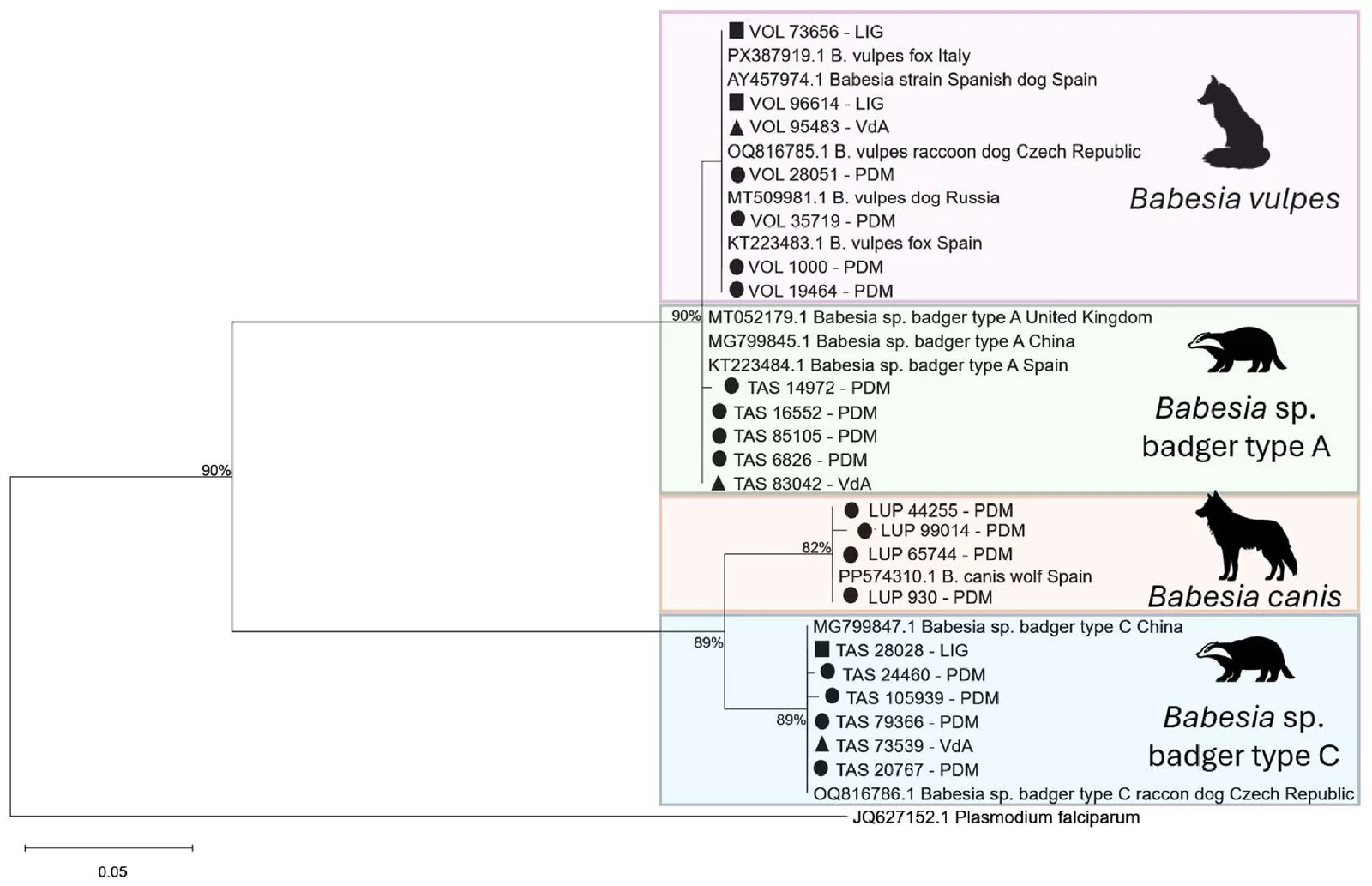
Maximum likelihood phylogenetic tree based on partial 18S rRNA sequences of *Babesia* spp. obtained from wild carnivores in northwestern Italy and reference sequences retrieved from GenBank. Bootstrap support values (≥70%) are shown at branch nodes. Sequences generated in the present study are indicated according to host species (LUP = wolf, VOL = fox, TAS = badger) and region of origin (● = Piemonte, ■ = Liguria and ▲ = Valle d’Aosta).

**Figure 3:**
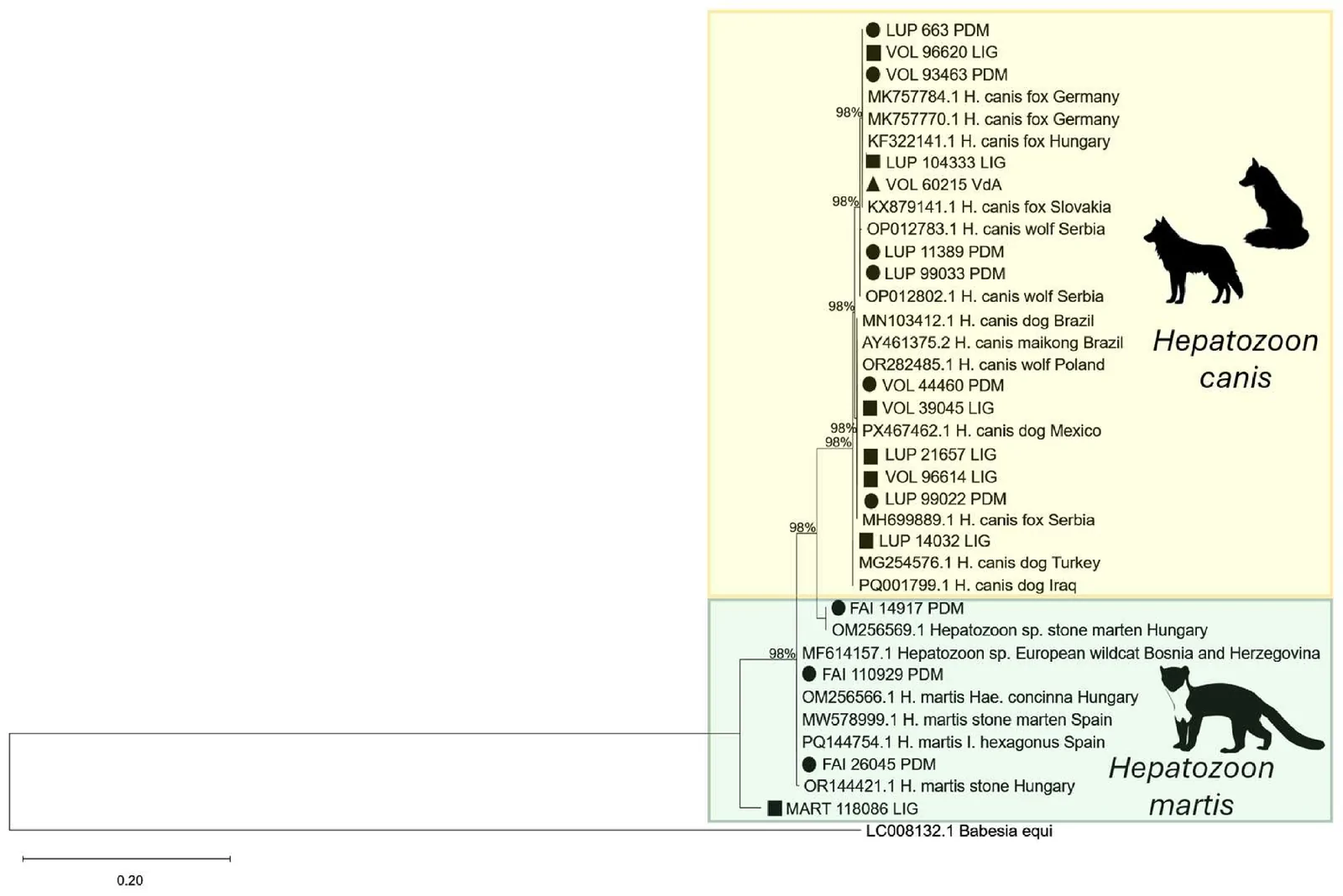
Maximum likelihood phylogenetic tree based on partial 18S rRNA sequences of *Hepatozoon* spp. obtained from wild carnivores in northwestern Italy and reference sequences retrieved from GenBank. Bootstrap support values (≥70%) are shown at branch nodes. Sequences generated in the present study are indicated according to host species (LUP = wolf, VOL = fox, FAI = stone marten, MART = pine marten) and region of origin (● = Piemonte, ■ = Liguria and ▲ = Valle d’Aosta).

**Figure 4:**
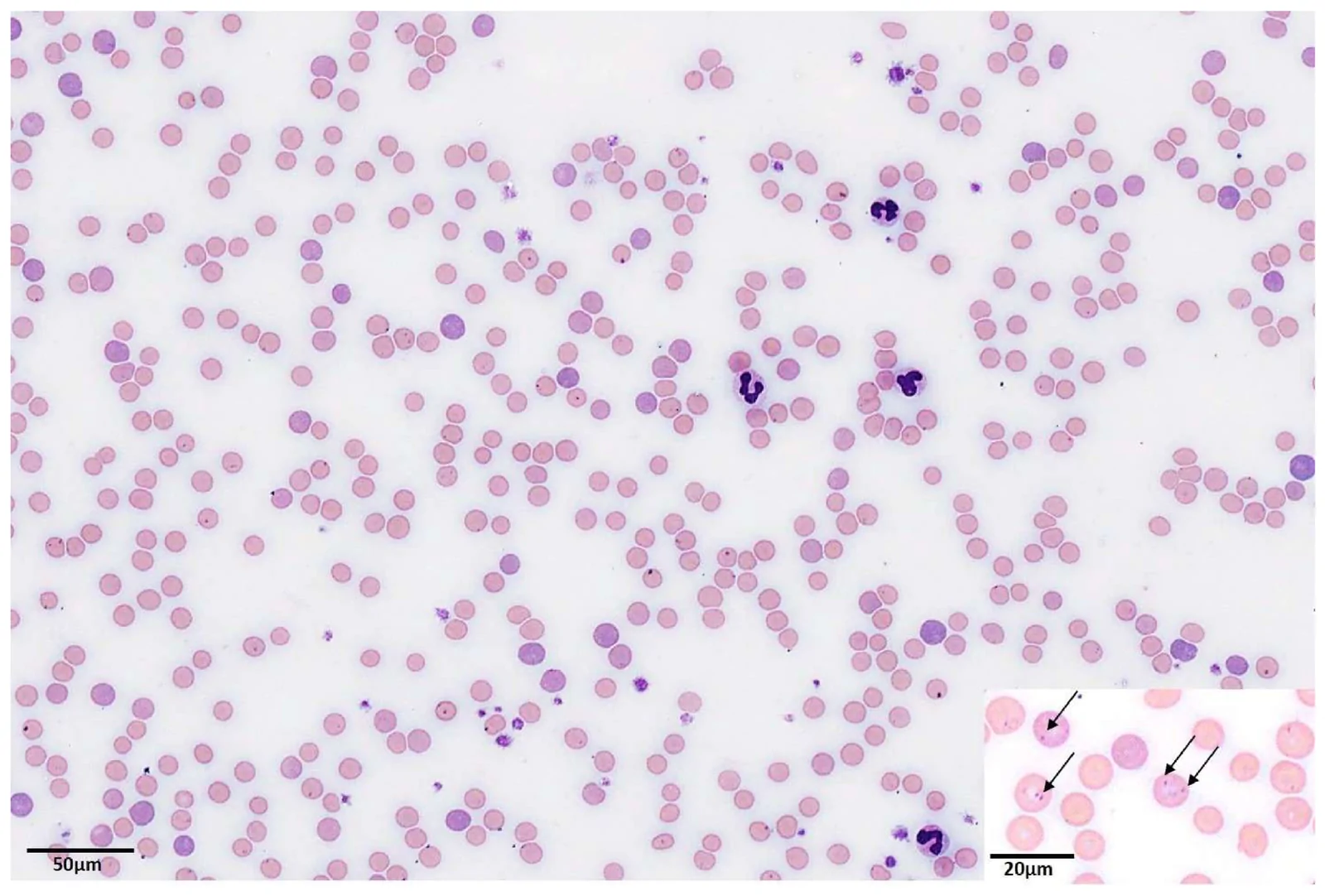
Blood smear from a *Babesia vulpes*-PCR-positive red fox showing intraerythrocytic piroplasms morphologically consistent with a small *Babesia* (arrows in inset). May-Grünwald Giemsa stain.

**Table 1:** Primers, target gene, fragment length, and PCR amplification conditions used for the molecular detection of *Babesia/Theileria* spp., *Hepatozoon* spp. and *Bartonella* spp.

Molecular Assay	Primers	Target Gene	Fragment Length	PCR Conditions	Reference
PCR for *Babesia* and *Theileria* species detection	Mic1 (GTCTTGTAATTGGAATGATGG) Mic2 (CCAAAGACTTTGATTTCTCTC)	18SrRNA	560 bp	95 °C–30 s 53 °C–30 s 72 °C–1 min (40 cycles)	[30] (slightly modified)
PCR for *Hepatozoon* species detection	HepF (ATACATGAGCAAAATCTCAAC) HepR (CTTATTATTCCATGCTGCAG)	18SrRNA	660 bp	95 °C–30 s 57 °C–30 s 72 °C–1 min (35 cycles)	[31]
PCR for *Bartonella* species detection	Nuo60 forward (GCGGCATAATTCGCATAACC) Nuo60 reverse (CACTTGGCAGTGCTATCCGTATT) NuoG60 probe (final concentration of 240 nM) (FAM-ACGACCCCGGCTAT-MGBEQ)	nuoG	346 bp	95 °C–10 s 60 °C–30 s (40 cycles)	[32]

**Table 2:** Population composition of wild carnivore species included in the study according to geographical origin, sex and age group.

	Canids n (%)		Mustelids n (%)				Total *n* (%)
	Wolf (*n* = 175)	Red Fox (*n* = 349)	European Badger (*n* = 132)	Stone Marten (*n* = 28)	Pine Marten (*n* = 5)	Otter (*n* = 1)	
Region							
Piemonte	126 (72.0)	229 (65.6)	87 (65.9)	13 (46.4)	4 (80.0)	0 (0.0)	459 (66.5)
Liguria	48 (27.4)	22 (6.3)	7 (5.3)	2 (7.2)	1 (20.0)	0 (0.0)	80 (11.6)
Valle d’Aosta	1 (0.6)	98 (28.1)	38 (28.8)	13 (46.4)	0 (0.0)	1 (100,0)	151 (21.9)
Sex							
Female	58 (33.1)	165 (47.3)	69 (52.3)	13 (46.4)	0 (0.0)	0 (0.0)	305 (44.2)
Male	99 (56.6)	170 (48.7)	59 (44.7)	13 (46.4)	4 (80.0)	1 (100)	346 (50.1)
Unknown	18 (10.3)	14 (4.0)	4 (3.0)	2 (7.2)	1 (20.0)	0 (0.0)	39 (5.7)
Age group							
Young	95 (54.3)	150 (43.0)	28 (21.2)	6 (21.4)	0 (0.0)	0 (0.0)	279 (40.4)
Adult	62 (35.4)	184 (52.7)	100 (75.8)	20 (71.4)	4 (80.0)	1 (100.0)	371 (53.8)
Unknown	18 (10.3)	15 (4.3)	4 (3.0)	2 (7.2)	1 (20.0)	0 (0.0)	40 (5.8)

Note: the ‘Young’ category includes cubs, juveniles and subadults. ‘Unknown’ indicates animals for which sex or age information was not available.

**Table 3:** PCR detection frequencies of *Babesia* spp., *Hepatozoon* spp. and *Bartonella* spp. according to wild carnivore host species.

Pathogen	Wolf	Red Fox	European Badger	Stone Martens	Pine Martens	Otter	Total
*Babesia* spp.	6/152 (3.9)	41/218 (18.8)	40/106 (37.7)	0/24 (0.0)	0/2 (0.0)	0/1 (0.0)	87 (17.3)
*Hepatozoon* spp.	70/152 (46.1)	46/218 (21.1)	0/106 (0.0)	3/24 (12.5)	1/2 (50.0)	0/1 (0.0)	120 (23.9)
*Bartonella* spp.	0/55 (0.0)	0/226 (0.0)	1/62 (1.6)	0/17 (0.0)	0/3 (0.0)	-	1 (0.3)

Note: Values are reported as the number of PCR-positive animals/number of animals tested (%). Pathogen-specific denominators differ because partially overlapping subsets of animals were screened for tick-borne protozoa and *Bartonella* spp. ‘-’ indicates that no animals of the corresponding host species were tested for that pathogen.

**Table 4:** Results of separate multivariable Firth penalized logistic regression models evaluating host- and region-related factors associated with *Babesia* spp. and *Hepatozoon* spp. positivity in wild canids from northwestern Italy.

Covariate	Category	*Babesia* spp.		*Hepatozoon* spp.	
		OR_adj_ (95% CI)	*p*-Value	OR_adj_ (95% CI)	*p*-Value
Species	Wolf (Ref.)	1.00		1.00	
	Fox	6.16 (2.55–17.70)	<0.001	0.41 (0.24–0.70)	<0.001
Age group	Young (Ref.)	1.00		1.00	
	Adult	0.57 (0.28–1.14)	0.112	0.61 (0.37–1.01)	0.056
Sex	Female (Ref.)	1.00		1.00	
	Male	0.83 (0.41–1.69)	0.609	1.34 (0.80–2.24)	0.263
Region	Piemonte (Ref.)	1.00		1.00	
	Liguria	1.03 (0.34–2.66)	0.960	0.51 (0.21–1.18)	0.115
	Valle d’Aosta	0.08 (0.01–0.31)	<0.001	0.31 (0.11–0.75)	0.008

Note: *n* = 337 complete cases for each model. OR_adj_: adjusted odds ratio; CI: confidence interval; separate Firth’s penalized logistic regression models were fitted for *Babesia* spp. and *Hepatozoon* spp. Reference categories were wolf for species, young for age group and Piemonte for region (OR = 1.00, Ref.). Age group was recategorized as “Young” (including cubs, juveniles and subadults) and “Adult”.

## Data Availability

The original contributions presented in this study are included in the article/Supplementary Materials. Further inquiries can be directed to the corresponding authors.

## Supplementary Materials

The following supporting information can be downloaded at: https://www.mdpi.com/article/10.3390/ani16192981/s1, Table S1. Detailed results per host species and pathogen (*Babesia* spp. and *Hepatozoon* spp.) according to the region of origin.

## Abbreviations

The following abbreviations are used in this manuscript:

IZSPLV	Istituto Zooprofilattico Sperimentale del Piemonte, Liguria e Valle d’Aosta
ML	Maximum Likelihood
NW	North-west
TBDs	Tick-borne diseases
TBP	Tick-borne protozoa
VBDs	Vector-borne diseases
VBPs	Vector-borne pathogens
