# Supplementary material for: Host-Specific Circulation and Molecular Diversity of Babesia, Hepatozoon and Bartonella Species Among Wild Carnivores in Northwestern Italy
Source: Animals (Basel). 2026 Sep 22;16(19):2981. doi: 10.3390/ani16192981 (PMC13663538; doi:10.3390/ani16192981)
Supplement: Supplementary file 1 [file animals-16-02981-s001.zip › animals-4513549-supplementary.pdf]

Table S1. Detailed results per host species and pathogen (*Babesia* spp. and *Hepatozoon* spp.) according to the region of origin

**Species**	**Number of positive (prevalence%)**							
	**Piedmont**		**Liguria**		**Aosta Valley**		**Total**	
	***Babesia* spp.**	***Hepatozoon* spp.**	***Babesia* spp.**	***Hepatozoon* spp.**	***Babesia* spp.**	***Hepatozoon* spp.**	***Babesia* spp.**	***Hepatozoon* spp.**
Red fox (*Vulpes vulpes*)	35/146 (23.97%)	35/146 (23.97%)	5/15 (33.33%)	6/15 (40%)	1/57 (1.75%)	5/57 (8.77%)	41/218 (18.81%)	46/218 (21.10%)
Wolf (*Canis lupus*)	6/116 (5.17%)	55/116 (47.41%)	0/35 (0%)	15/35 (42.86%)	0/1 (0%)	0/1 (0%)	6/152 (3.95%)	70/152 (46.05%)
European badgers (*Meles meles*)	33/69 (47.83%)	0/33 (0%)	4/5 (80%)	0/5 (0%)	3/33 (9.09%)	0/33 (0%)	40/107 (37.38%)	0/107 (0%)
Stone martens (*Martes foina*)	0/9 (0%)	3/9 (33.33%)	0/2 (0%)	0/2 (0%)	0/12 (0%)	0/12 (0%)	0/23 (0%)	3/23 (13.04%)
Pine martens (*Martes martes*)	0/1 (0%)	0/1 (0%)	0/1 (0%)	1/1 (100%)	/	/	0/2 (0%)	1/2 (50%)
Otter (*Lutra lutra*)	/	/	/	/	0/1 (0%)	0/1 (0%)	0/1 (0%)	0/1 (0%)
